# HER2 gene and protein expression status of breast carcinoma can be reliably tested on a single slide

**DOI:** 10.1007/s00428-015-1781-0

**Published:** 2015-05-19

**Authors:** Marie-Pierre Chenard, Marie-Pierre Wissler, Noëlle Weingertner, Carole Mathelin, Jean-Pierre Bellocq

**Affiliations:** Department of Pathology, University Hospitals of Strasbourg, 1 avenue Molière, 67098 Strasbourg, France; Department of Gynecology and Obstetrics, University Hospitals of Strasbourg, Strasbourg, France

**Keywords:** HER2, Breast carcinomas, 4B5, SISH, Gene, Protein expression, Simultaneous

## Abstract

Human epidermal growth factor receptor 2 (HER2) status in breast carcinomas serves as a predictor of benefit from anti-HER2 therapy. In providing clinicians with the information necessary to decide whether or not to treat with targeted therapy, it might be necessary to choose between methods assessing HER2 protein overexpression or gene amplification. A new diagnostic approach could be a combination of both tests on the same slide. If accurate and reproducible, this approach might optimize patient stratification for therapy. In this study, formalin-fixed paraffin-embedded tumor samples from 77 breast cancer patients were examined for HER2 by immunohistochemistry (IHC) and silver in situ hybridization (SISH) using HER2 IHC (clone 4B5), HER2/CEN17SISH, and combined IHC and SISH assay, called gene protein (GP). Cases were selected to ensure a sufficient number of borderline cases on the basis of IHC scores (0, 1+, 2+, 3+), obtained during diagnostic histopathological workup. The concordance between the HER2 IHC score obtained during diagnostic histopathological workup and GP was 93 %. Discordances had no influence on therapy decisions. The concordance between ISH results using dual ISH (DISH) and GP was 96 %. Of the 77 cases studied by GP, three cases with a ratio close to 2 would have been called amplified by DISH. The use of GP reduced the time for slide reading for a trained pathologist by up to 25 %, relative to sequential reading of IHC followed by SISH. For cases with an IHC score of 2+, the final result was obtained in 1 day, while the sequential technique would have required retesting by ISH on a second day. In conclusion, assessment of HER2 status by GP is an improvement for pathologists and facilitates clinical decision-making for breast cancer management.

## Introduction

Human epidermal growth factor receptor 2 (HER2) is a protein on the surface of cancer cells that stimulates tumor growth. When the *HER2* gene is amplified, it triggers overproduction, commonly called overexpression, of HER2 [1] protein. Tumors that strongly overexpress HER2 and/or those with a proven amplification of the *HER2* gene are classified as being HER2 positive. HER2-positive cancers are associated with poor overall prognosis with faster time to relapse or progression at all stages [2–4]. In the early days of HER2 testing, amplification of the *HER2* gene and the corresponding overexpression of HER2 protein was found in approximately 25 to 30 % of breast cancer [5], but this rate was probably an overestimate as it is now identified in approximately 15 to 20 % [6] of primary breast cancer cases, while recent data show a further decreasing trend to around 14 % [7].

Assessment of HER2 status in the breast is required to support treatment decisions, as it predicts response to HER2 targeted therapies. Presently, four HER2-targeted therapies are approved by the Food and Drug Administration (FDA) for treatment of HER2-positive breast cancer: Trastuzumab^®^, Lapatinib^®^, Pertuzumab^®^, and Trastuzumab emtasine^®^. In addition, promising new approaches are being developed including monoclonal antibodies and small-molecule tyrosine kinase inhibitors targeting HER2 or other HER family members, antibodies linked to cytotoxic moieties or modified to improve their immunological function, immunostimulatory peptides, and PI3K and IGF-1R pathway [8, 9] targets.

To date, only two techniques for HER2 status determination are validated, FDA approved, and broadly used in a diagnostic setting. Immunohistochemical (IHC) analysis identifies HER2 protein expression on the cell surface, while in situ hybridization (ISH) determines the degree of HER2 gene amplification. Both methods are highly specific and reproducible when performed under standardized and validated conditions. HER2 testing has been standardized for breast carcinoma, and the American Society of Clinical Oncology (ASCO) recommends that HER2 status should be determined for all invasive breast cancers [10, 11]. The ASCO/CAP 2007 HER2 guidelines provide an algorithm defining positive and negative status for both HER2 protein expression and gene amplification. Cases demonstrating IHC staining of 3+ (uniform, complete intense membranous staining of more than 30 % of invasive tumor cells) or an ISH HER2 copy number ≥6 or a ratio HER2 gene signal to chromosome 17 signal ≥2.2 are considered positive. A negative result is IHC staining of 0 or 1+ or ISH HER2 copy number <4 or a ratio <1.8. The recently revised ASCO-CAP 2003 guidelines state that all tumors with complete, intense circumferential membrane staining of more than 10 % of the cells are considered as 3+ and therefore positive. HER2 status by ISH is positive for cases showing a HER2 gene to chromosome 17 signal ratio ≥2, regardless of the number of HER2 copies. Cases with a HER2 gene to chromosome 17 ratio <2 but with a HER2 copy number of 6 or higher are also considered positive.

Cases with complete membrane staining, that is either nonuniform or weak in intensity but with obvious circumferential distribution in at least 10 % of cells (IHC 2+), are considered equivocal and require assessment by ISH [19]. This group accounts for approximately 15 % of all tumors and, for optimal guidance of HER2 targeted therapy, further quantification of gene copy number to determine amplification status is needed [12]. In the revised version of the ASCO-CAP guidelines, the definition of IHC equivocal cases changed to tumors with circumferential membrane staining that is incomplete and/or weak/moderate in more than 10 % of tumors cells, or with intense complete circumferential membrane staining in less than or equal to 10 % of the tumor cells.

For cases with an equivocal ISH ratio of 1.8-2.2, additional cells should be scored to allow a diagnostic decision to be reached. A ratio of 1.80-1.99 should be reported as borderline not amplified and HER2 negative and a ratio of 2.00-2.20 as borderline amplified and HER2 positive. It is recommended that laboratories show at least 95 % concordance between IHC and ISH. The revised version of the ASCO-CAP guidelines also changes the definition for equivocal cases defined by ISH testing. Cases with an HER2 gene copy to chromosome 17 signal ratio above 2 or with an HER2 signal number between 4 and 6 are considered equivocal.

Based on the ASCO guidelines, the currently most widely used testing algorithm for breast carcinoma consists of first-line HER2 IHC staining followed by ISH testing for 2+ cases. This algorithm may result in some discordant cases, e.g., IHC 0 or 1+ cases amplified in ISH or IHC 3+ cases nonamplified in ISH, amounting to about 4 % of cases as described by Lee et al. [13] and Bernasconi et al. [14]. In addition, intratumor heterogeneity of HER2 gene amplification has been observed in breast cancer, ranging from a few [15] to up to 36 % of the amplified cases [16], which introduces additional difficulties in HER2 status evaluation especially for equivocal cases [17]

The long history of HER2 testing in breast cancer is an indication of the importance of continuing efforts to improve HER2 diagnostic assays and their interpretation, to ensure that patients who may benefit receive the appropriate targeted therapy. A new robust and reliable method, that would allow assessment of protein expression and gene amplification status on the same slide, could be a significant improvement. The aim of this study is to test the feasibility and reliability of this new method focusing especially on borderline breast cancer cases.

## Materials and methods

### Study design

A new gene and protein detection platform (GP, Ventana) combines IHC staining with ISH. To estimate HER2 GP reliability, results were compared with FDA-approved INFORM HER2 DISH DNA Probe Cocktail and HER2 IHC (clone 4B5) assays (Ventana).

### Patients

Patients were selected based upon previously performed diagnostic IHC staining, with the intention to include a sufficient number of borderline cases. This resulted in a case series of 10 cases without IHC overexpression (0), 18 with weak staining (1+), 35 with moderate staining (2+), and 14 with strong staining (3+). For this study, HER2 IHC status of all cases was confirmed by a second IHC analysis done in one staining run.

### Immunohistochemical analysis

Formalin-fixed paraffin-embedded sections were dried at 60 °C for 2 h and then processed on a BenchMark XT automated slide-stainer (Ventana). All used reagents were from Ventana. After deparaffinization and pretreatment (Cell Conditioning I for 30 min at 95 °C), sections were incubated with primary antibody (clone 4B5) for 20 min at 37 °C and secondary antibody (HRP-conjugated goat anti-rabbit antibody). Immunoreactivity was detected with the iView DAB Detection Kit, and counterstaining was performed with Hematoxylin II.

### Dual color in situ hybridization

Formalin-fixed paraffin-embedded sections were dried at 60 °C for 2 h and then processed on a BenchMark XT automated slide-stainer (Ventana). All used reagents were from Ventana. After deparaffinization and pretreatment (Cell Conditioning II, protease, denaturation), sections were exposed to probe hybridization (Chromosome 17 centromere labeled with DIG and HER2 labeled with DNP) with HybReady and stringency washing using SSC solution. The DNP probe was detected with *ultra*View SISH DNP [black dots] and the DIG probe with the *ultra*View Red [red dots], and the slides were counterstained with hematoxylin.

### HER2 gene and protein platform

Formalin-fixed paraffin-embedded sections were dried at 60 °C for 2 h and then processed on a BenchMark XT automated slide-stainer (Ventana). All used reagents were from Ventana. The staining procedure in essence consisted of subsequent application of the IHC and double ISH procedures as described above. HybClear is a unique reagent specific to the Ventana GP platform that enables combination of both techniques on a single slide by blocking silver dust background. The total duration of the protocol was about 15 h.

### Scoring of staining results

IHC and ISH results were scored by an experienced breast pathologist (MPC) only in invasive carcinoma, by bright field microscopy using ×20, ×40, and ×60 objectives. IHC staining results were classified according to ASCO-CAP 2007 guidelines taking into account staining intensity, localization, and extent (% of positive staining cells) into 0, 1+, 2+, and 3+ groups. ISH signals were reported as single copies, multiple copies, and clusters. Normal HER2 or CEN17 signals (one to two copies/cell in stromal fibroblasts, endothelial cells, lymphocytes, and benign breast epithelial cells) served as internal positive control, which was considered as adequate when the signals were visible in the sample at any magnification used. A minimum of 10 cells were scored in cases without variation between nuclei or clustered signals, and 20 to 40 cells were scored for cases with 2+ IHC and/or internuclear variation. The number of HER2 signals was divided by the number of CEN17 signals to obtain a HER2/CEN17 ratio.

GP-stained slides were first evaluated at low power to score HER2 expression, as performed for only IHC-stained slides. An ISH score was then established at higher magnification. Slides were read in a randomized manner by session of 45 slides (15 IHC, 15 ISH, and 15 GP slides). To avoid influence of previously seen slides on scoring, within a reading session IHC, ISH, and GP were independently scored and subsequent sessions never concerned the same 15 cases. The duration of scoring was measured for each category of slides using 0.5-min increments.

## Results

### Visual appreciation

Single IHC staining was successful on all cases. Dual SISH was successful initially on 69 cases and with an additional staining run on 3 more cases. GP staining was successful on 74 cases after the first run and on 3 more cases with a second run. This left 72 cases for which results of the 3 staining procedures were available.

**Fig. 1:**
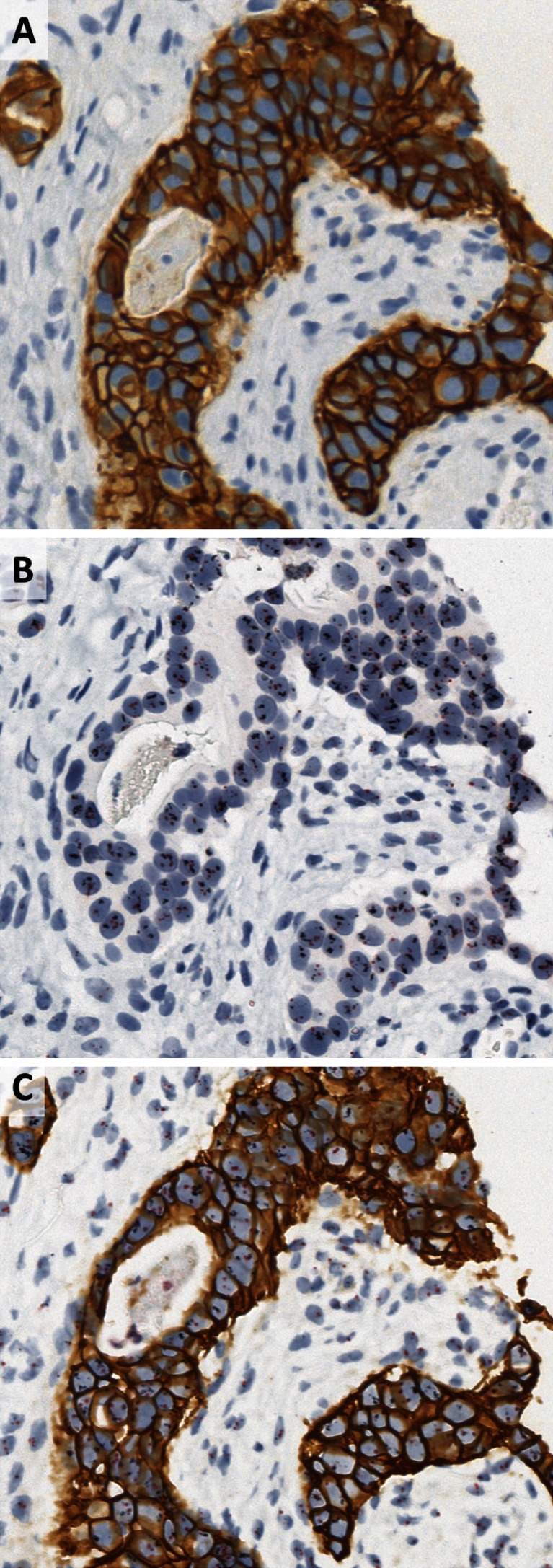
IHC (**a**), DISH (**b**), and GP (**c**) staining of the same breast carcinoma case at ×40. HER2 IHC score is determined as 3+ for both IHC and GP, with similar staining pattern and intensity. Similar amplification with clusters is observed in both DISH and GP techniques

The first impression when looking at GP-stained slides was that immunostaining is less crisp, which might be due to the pretreatment required for the DISH step. However, this had no impact on reading or scoring, following ASCO-CAP 2007 or the recently published ASCO-CAP 2013 guidelines, although reading GP-stained slides might require some time for an experienced pathologist to adapt (Fig. 1). The quality of DISH staining obtained with GP was judged as good as the with DISH staining only. In some HER2 3+ cases, DISH staining resulted in smaller dots or clusters, or areas of strong membranous DAB staining without ISH signals. In these cases, IHC 3+ score and/or an HER2/CEN17 ratio of 2 (or more than five copies of HER2) in other areas allowed valid HER2 status assessment (Fig. 1).

### Concordance of IHC on single and GP-stained slides

The concordance between HER2 expression using IHC only and GP was 93 % (Table 1). Minor discrepancies were observed for 1+ cases, without any impact on HER2 status assessment.

**Table 1:** Comparison of HER2 IHC score determined on IHC and GP slides

		IHC			
		0	1+	2+	3+
GP	0	7	4	0	0
	1+	1	11	0	0
	2+	0	0	36	0
	3+	0	0	0	13

### Concordance of ISH on DISH and GP-stained slides

The concordance between the HER2/CEN17 ratios obtained on DISH and GP-stained slides was 94 % (96 % when reduced to amplified or not amplified status, Tables 2 and 3). In three cases, DISH and GP results were discrepant. These were borderline cases, corresponding to those reported in the literature with interobserver and intraobserver variability (HER2/CEN17 between 1.8 and 2.2).

**Table 2:** Comparison of HER2 IHC score determined on IHC and GP slides classified as NEG (negative) for 0 and 1+ and POS (positive) as 2+ and 3+ cases

		IHC	
		NEG	POS
GP	NEG	23	0
	POS	0	49

**Table 3:** Comparison of HER2 ISH score determined on DISH and GP slides classified as NEG (negative) when case show a ratio HER2/Chromosome 17 below 2 and less than six copies and POS (positive) when the case show a ratio above 2 or a mean copy number of HER2 gene above or equal 6

		DISH	
		Neg	Pos
GP	Neg	46	2
	Pos	1	23

### Concordance of HER2 status on IHC + ISH and GP-stained slides

**Fig. 2:**
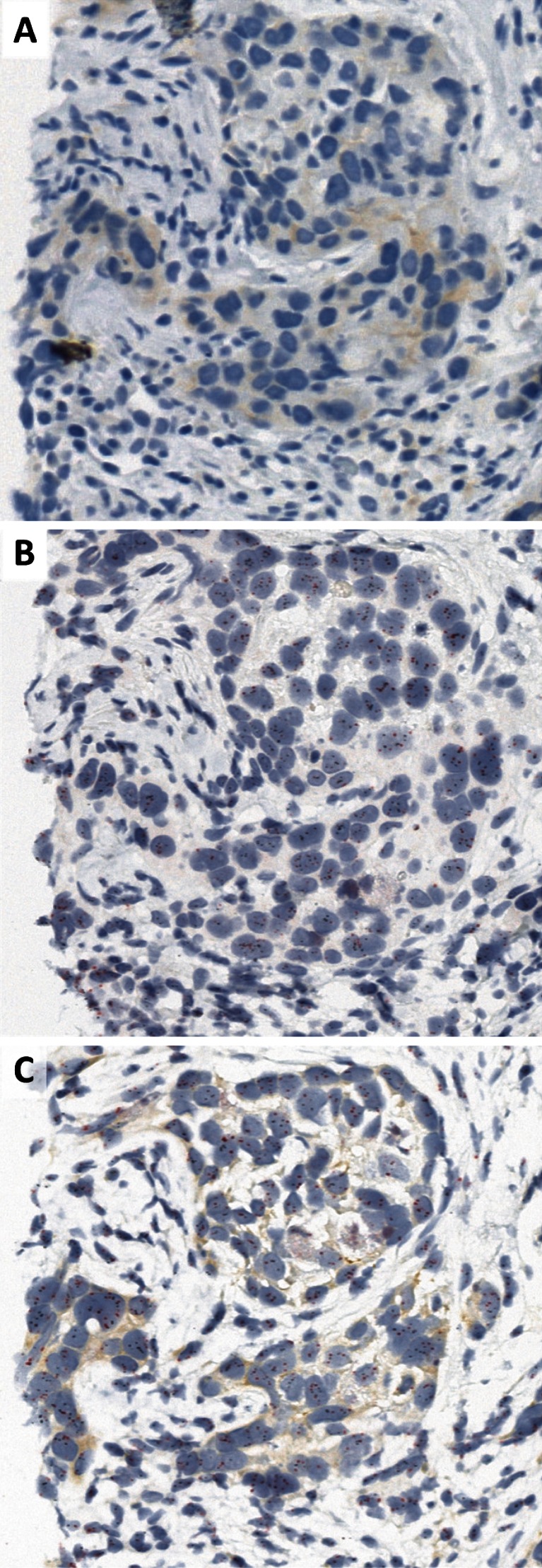
IHC (**a**), DISH (**b**), and GP (**c**) staining of the same breast carcinoma case at ×40. HER2 IHC score is determined as 0 for both IHC and GP, with similar staining pattern and intensity (faint cytoplasmic). Low level amplification was detected (≥6 copies, ratio above 2 due to minor polysomy) in both DISH and GP slides. This case would not have been eligible for HER2-targeted therapy using IHC alone

Using the previously described algorithm (first HER2 IHC and DISH only for 2+ cases), with one exception all cases were concordant (Table 4). The discordant case was scored as 0 by IHC in single staining IHC and GP but ISH amplified by both DISH and GP staining (mean HER2 copy number >6, HER2/CEN17 <2 due to polysomy) (Fig. 2), even though close to the cutoff value (6.5 for DISH and 6.05 for GP).

**Table 4:** Comparison of HER2 status using combination of HER2 IHC and DISH techniques vs. GP

		IHC + DISH	
		Neg	Pos
GP	Neg	48	0
	Pos	1	23

Positive and negative cases are defined according to ASCO-CAP guidelines

### Reading time comparing IHC + ISH with GP

The regression curve comparing reading time to result of IHC followed by DISH with GP showed *y* = 0.7355, corresponding to a gain in time of reading of 25 %, notably when focusing on borderline (1+ and 2+ IHC) cases (data not shown).

## Discussion

In this study, we tested a new technique for simultaneous assessment of HER2 protein expression and gene amplification status. This technique was first described by Nitta et al. [18] and Hierschmann et al. [19] and optimizes determination of HER2 status as well as allowing identification of cases in which HER2 IHC and HER2 ISH statuses are not concordant. Although breast cancer heterogeneity has been described as a major cause of variability in the assessment of HER2 status, this was not the focus of our study. The possibility to observe simultaneously HER2 expression and HER2 amplification will guide the observer to the most relevant tumor area and also show areas in which HER2 amplification is not accompanied by protein overexpression. Such differences might be related to preanalytical parameters or to as-yet unidentified biological events [20] .

Although IHC and ISH are robust and reliable techniques, misclassification does occur following the ASCO-CAP testing algorithm [14], which might incur changes in patient treatment. Only patients with a positive HER2 status, either HER2 IHC 3+ or amplified, will be treated with one of the anti HER2 therapies [23–27]. In our study, one case did not show overexpression (scored as 0 both in IHC only and GP) but was amplified (in both techniques, Fig. 2). This case would have been classified as negative using the ASCO-CAP testing algorithm. However, as the case showed more than six copies of HER2 although it was polysomic (ratio of HER2/Chromosome 17 below 2), following the same ASCO-CAP rules this patient qualifies for treatment with targeted anti-HER2 therapy.

We found the GP assay more robust than the DISH assay. After GP staining, 96 % of the slides could be scored while this was the case for only 90 % of the DISH-stained slides. This may be due to the stronger pretreatment necessary in GP staining as it consists of successive pretreatments for IHC and for DISH (the latter being similar to DISH only but longer and with a higher protease concentration).

In our institution, the workflow for HER2 testing of IHC 2+ cases requires up to 3 days because of the need for additional ISH testing. This is acceptable but may become problematic when due to technical failure a second staining round would be required especially for ISH, as in such event a final result for HER2 status assessment might take up to 1 week. Using GP, complete HER2 status can be determined in 1 day, or 2 days if the staining is done overnight. Moreover, although the success rate of GP is not perfect, it was better than ISH alone, with only three cases that could not be interpreted after a single staining round. Workflow is also positively affected by the use of only a single slide. However, the cost of the GP test is higher than that of the two separate IHC and DISH tests, and therefore, assessment of cost-effectiveness should be conducted to arrive at a comprehensive cost-benefit analysis in terms of treatment optimization [21, 22] .

For borderline cases, we observed that GP reduced the time to get to a final result by 25 %. The reading time required for evaluation of 0 and 3+ cases is not changed in the GP assay, as HER2 protein expression of these cases will be read at low magnification. However, evaluating protein expression and gene amplification in the same slide allows the pathologist to avoid false negative such as in our case with HER2 IHC 0 but HER2 amplification.

The number of tests required to arrive at a final diagnosis increases constantly and will continue to do so in the coming years with new targeted therapies coming along. More tests will require more biological materials, while at the same time clinicians attempt to decrease invasive diagnostic procedures such as surgery or biopsy, which confronts pathologists with reduced sample size. Sample size may become too low to do all required tests with traditional approaches. Consequently, combined testing will be increasingly important to get as much information as possible from little material. GP combines assessment of gene amplification with that of protein expression and provides an elegant option for assessment of HER2 status in breast cancer. As a generic detection method, GP is not limited to one biomarker and provides an opportunity for exploring other marker tests in other diseases.

In conclusion, the combined GP assay improves laboratory workflow and improves patient management by delivering more precise and faster results. A final result for HER2 2+ cases can be delivered within 1 day, while sequential staining requires at least a second day to perform subsequent SISH testing. Efficacy could be further increased by a higher success rate, which needs to be confirmed in further studies.
